# Serum uric acid levels and risk of kidney cancer incidence and mortality: A prospective cohort study

**DOI:** 10.1002/cam4.3214

**Published:** 2020-06-14

**Authors:** Xiao‐Yu Dai, Qiang‐Sheng He, Zhong Jing, Jin‐Qiu Yuan

**Affiliations:** ^1^ Department of Nephrology Mianyang Central Hospital Mianyang China; ^2^ Clinical Research Center The Seventh Affiliated Hospital Sun Yat‐Sen University Shenzhen Guangdong China; ^3^ Scientific Research Center The Seventh Affiliated Hospital Sun Yat‐Sen University Shenzhen Guangdong China; ^4^ Department of Nephrology Mianyang 404 Hospital Mianyang China

**Keywords:** kidney cancer, prospective study, UK Biobank, uric acid

## Abstract

**Objective:**

Epidemiological evidence investigating serum uric acid and kidney cancer risk remains unclear. We conducted this study to examine the relationship between serum uric acid and the incidence and mortality of kidney cancer.

**Methods:**

This is a prospective analysis of 444 462 participants without any cancer from the UK Biobank. Serum uric acid was measured at baseline and the incidence and mortality of kidney cancer was determined through contact with the cancer and death registry. Cox regression models were fitted to estimate the hazard ratio (HR) and 95% confidence interval (95%CI), adjusting for demography, lifestyle style, comorbidities, and medication use.

**Results:**

We documented 638 incidence cases and 188 mortality cases of kidney cancer over a median of 6.5 years follow‐up. People with the highest quartile had a 45% increased risk of kidney cancer compared to those with the lowest uric acid quartile (HR 1.45, 95%CI 1.08 to 1.93). Subgroup analyses showed that serum uric acid was associated with cancer risk among females but not among males (Q1 vs Q4: females HR1.47, 95%CI 1.01 to 2.16; males HR 1.19, 95%CI 0.91 to 1.56). Although we found serum uric acid was associated with an increased risk of kidney cancer mortality in age‐stratified model (HR 2.49, 95% CI 1.61 to 3.84), this association disappeared after further adjustment for other confounders.

**Conclusions:**

High uric acid is associated with a high incidence of kidney cancer, especially in women. More research is needed to confirm our findings.

## 1 INTRODUCTION

Worldwide there are more than 330,000 new cases of kidney cancer diagnosed each year, accounting for 2.4% of all cancers.^1^ In developed regions such as Europe, North America, Australia/New Zealand, and Japan, kidney cancer is the seventh most common cancer. The incidence of kidney cancer has been increasing globally in the last 50 years.^2^ In most countries, the average annual growth rate is about 2% to 3%.^3^ The most common subtype of kidney cancer is clear cell renal cell carcinoma (ccRCC), which accounts for 75% of all cases. Patients with metastatic ccRCC have a poor prognosis, with a 5‐year survival rate of < 10%. Many risk factors for kidney cancer have been identified, including smoking, obesity, hypertension, and chronic kidney disease.

Serum uric acid (SUA) is an antioxidant that plays an important role in scavenging free radicals and is rich in the blood.^4^ Previous studies have linked SUA to cancer risk. In 2019, a meta‐analysis of 24 articles showed the hyperuricemia was associated with an increased risk of cancer in men (Risk Ratio [RR]1.07; 95% Confidence Interval [CI] 1.03‐1.11) and increased mortality in women (RR 1.26; 95% CI 1.09 to 1.45).^5^ However, studies investigating the relationship between SUA and kidney cancer are still scarce. To date, only one study suggested that people with hyperuricemia had higher risk of kidney cancer (Q4 vs Q1, Hazard Ratio [HR] 1.27, 95% CI 1.06 to 1.51).^6^ However, this study was carried out in a selection of a healthy population and the result was limited due to insufficient control for important confounding factors, such as alcohol intake and smoking. Further investigation of the association between SUA to kidney cancer is still required.

The UK biobank is an ongoing prospective cohort of UK residents. This cohort has collected a wide range of information on socio‐demographic factors, lifestyle factors and biomarkers, and health outcomes. In our study, we examined the relationship between SUA and the risk and mortality of kidney cancer using the UK biobank dataset.

## 2 METHODS

### 2.1 Research and participants

UK Biobank is a well‐characterized prospective cohort study of over 0.5 million participants aged 40‐69 years. The participants were recruited throughout England, Wales, and Scotland during 2006‐2010. All included participant completed a touchscreen questionnaire and provided physical measures and biological samples at baseline assessment. Details about the study design and assessments of the UK Biobank cohort have been published elsewhere.^7^ The UK Biobank study has been approved by the North West Multi‐center Research Ethics Committee. Prior to data collection, all participants provided written informed consent.

The UK Biobank dataset included 502 527 participants. For our analysis, participants were excluded if they had any cancer diagnosis prior to baseline (except for nonmelanoma skin cancer ICD‐10 C44) (n = 26 868), had missing data on measure of SUA (n = 31 197). Finally, we included 444 462 participants in the present analysis.

### 2.2 Assessment of outcome

Participants in the UK biobank were tracked in the Health and Social Care Information Centre (in England and Wales) and the National Registry of Health Services (in Scotland), which provides information about cancer. Cancer was coded by the 10th revision of the international classification of diseases (ICD‐10) in these registries. The endpoints in this study are the first diagnosis of kidney cancer (ICD‐10 codes C64) and kidney cancer caused death. At the time of analysis, mortality data were available up to 31 November 2016 and cancer registration data were available up to 30 October 2015. Person‐years of follow‐up were calculated from date of the recruitment date to the date of death, the last date of follow‐up, or the date of first diagnosis of cancer (except nonmelanoma skin cancer, ICD‐10 C44).

### 2.3 Assessment of exposure

SUA was measured by uricase PAP analysis on a Beckman Coulter AU5800. SUA levels were categorized into quartiles. In the subgroup analyses by sex, SUA was categorized by sex quartile. In addition, according to the equivalence, the SUA value expressed in μmol/L was converted to the value expressed in mg/dL for comparison with other research results (1 mg/dL = 59.55 μmol/L).

### 2.4 Covariates

Covariates were collected from participant interview at the recruitment. The covariates included age, sex, education (*the highest qualification achieved*), ethnic (*white/non‐white*), index of multiple deprivation quintile (*fifth, a measure of socioeconomic status*), comorbidities (*hypertension, diabetes, renal failure, and gout*) and medication use (*cholesterol lowering medication, blood pressure medication, and NSAIDS*). Lifestyle factors including alcohol consumption (*Daily or almost daily, one to four times a week, one to three times a month, only for special occasions or never*), smoking status (*never, current, and previous*), physical activity (*metabolic equivalents [METs] score was calculated and divided into three groups: low, moderate, and high*), and portions of fruit and vegetable intake per day. Body mass index (BMI) in kg/m was divided into four categories (under [<18.5], normal weight [18.5‐25], overweight [25 to < 30], or obese [30 or higher]) based on height and weight measurements recorded at baseline.

### 2.5 Data analysis

We used an age‐based Cox regression model (37‐49 years, 50‐59 years, ≥75 years) to estimate the HR and 95% CI between uric acid and the risk and mortality of kidney cancer. In the multivariable‐adjusted model 1, we adjusted for gender, education, ethnic, and index of multiple deprivation. To control the potential confounding from lifestyle factors, we additionally adjusted for alcohol consumption, smoking status, physical activity, and fruit and vegetable intake in the multivariate adjustment model 2. In the multivariate adjustment model 3, we additionally controlled for body mass index (BMI), comorbidities (diabetes, hypertension), and medication (cholesterol lowering medication, blood pressure medication, and NASIDS). We created a response “unknown/missing” category for covariates where participants chose “don't know” or “don't want to answer” or missing data.

We performed a number of sensitivity analyses to check the robustness of the primary results. First, to minimize potential reverse causation (ie, SUA concentrations being affected by undiagnosed kidney cancer), we excluded the participants who developed kidney cancer during the first 2 years of follow‐up. Second, we excluded participants with renal failure to reduce the potential influence. Last, we limited the analysis in people without gout to reduce the influence of gout disease. We used R software for all analyses (version 3.5.0, R Foundation for Statistics Computing).

## 3 RESULTS

A total of 238 856 women and 205 606 men were included in the study. The baseline characteristics of included participants by quartiles of SUA are shown in Table 1. The participants in the top quartile of SUA (Q4) were more likely to be male, obese, previous smokers, have higher alcohol consumption and lower consumption of fruit and vegetable, have a higher rate of hypertension, diabetes, and gout, and were more likely to use cholesterol lowering medication, and blood pressure medication.

**TABLE 1:** Participants characteristics according to baseline serum uric acid levels

	Quartile of serum uric acid level			
	Quartile 1	Quartile 2	Quartile 3	Quartile 4
Number of participants	111 087	110 975	111 241	111 159
Follow‐up years	6.58 (1.17)	6.55 (1.22)	6.53 (1.24)	6.51 (1.29)
Mean (SD) age, y	54.8 (8.20)	56.4 (8.01)	57.0 (7.98)	57.2 (8.01)
Gender				
Female	99 225 (89.3%)	73 883 (66.6%)	44 282 (39.8%)	21 466 (19.3%)
Male	11 862 (10.7%)	37 092 (33.4%)	66 959 (60.2%)	89 693 (80.7%)
N (%)White	104 975 (94.5%)	104 411 (94.1%)	104 447 (93.9%)	104 475 (94.0%)
Index of multiple deprivation Quintile				
First	19 873 (17.9%)	19 448 (17.5%)	19 156 (17.2%)	18 164 (16.3%)
Second	19 776 (17.8%)	19 218 (17.3%)	19 179 (17.2%)	18 476 (16.6%)
Third	19 109 (17.2%)	19 158 (17.3%)	19 008 (17.1%)	18 890 (17.0%)
Fourth	18 635 (16.8%)	19 012 (17.1%)	19 096 (17.2%)	19 487 (17.5%)
Fifth	17 492 (15.7%)	18 366 (16.5%)	19 359 (17.4%)	20 548 (18.5%)
Smoking status				
Current	11 629 (10.5%)	11 795 (10.6%)	12 154 (10.9%)	11 444 (10.3%)
Previous	31 872 (28.7%)	35 290 (31.8%)	39 277 (35.3%)	45 874 (41.3%)
Never	67 091 (60.4%)	63 339 (57.1%)	59 221 (53.2%)	53 244 (47.9%)
Alcohol consumption				
Daily or almost daily	17 394 (15.7%)	20 290 (18.3%)	23 736 (21.3%)	29 009 (26.1%)
One to four times a week	52 788 (47.5%)	53 907 (48.6%)	55 027 (49.5%)	55 826 (50.2%)
One to three times a month	14 577 (13.1%)	13 324 (12.0%)	11 850 (10.7%)	9681 (8.7%)
Special occasions only or never	26 102 (23.5%)	23 203 (20.9%)	20 363 (18.3%)	16 388 (14.7%)
Physical activity				
Low	14 838 (13.4%)	16 003 (14.4%)	17 162 (15.4%)	19 457 (17.5%)
Moderate	37 562 (33.8%)	36 235 (32.7%)	36 318 (32.6%)	36 115 (32.5%)
High	36 219 (32.6%)	36 706 (33.1%)	36 948 (33.2%)	36 029 (32.4%)
Mean (SD) fruit and vegetable intake, portions per day	4.93 (3.12)	4.76 (3.16)	4.52 (3.11)	4.25 (3.03)
BMI				
Low weight	1438 (1.3%)	502 (0.5%)	214 (0.2%)	88 (0.1%)
Normal	59 992 (54.0%)	41 242 (37.2%)	27 793 (25.0%)	15 202 (13.7%)
Overweight	37 310 (33.6%)	46 918 (42.3%)	52 408 (47.1%)	51 908 (46.7%)
Obesity	11 947 (10.8%)	21 887 (19.7%)	30 379 (27.3%)	43 461 (39.1%)
Comorbidities (%)				
Hypertension	17 915 (16.1%)	24 911 (22.4%)	32 044 (28.8%)	43 887 (39.5%)
Diabetes	4039 (3.6%)	5130 (4.6%)	6070 (5.5%)	7752 (7.0%)
Renal failure	75 (0.1%)	111 (0.1%)	154 (0.1%)	395 (0.4%)
Gout	639 (0.6%)	1038 (0.9%)	1237 (1.1%)	3350 (3.0%)
Medication use				
NSAIDS	43 688 (39.3%)	43 672 (39.4%)	43 742 (39.3%)	45 911 (41.3%)
Cholesterol lowering medication	10 937 (9.8%)	16 153 (14.6%)	21 111 (19.0%)	28 372 (25.5%)
Blood pressure medication	11 976 (10.8%)	18 168 (16.4%)	24 794 (22.3%)	36 467 (32.8%)

Abbreviation: BMI, body mass index; NSAIDs, nonsteroidal anti‐inflammatory drugs; SD, standard deviation.

### 3.1 Incidence of kidney cancer

With a median follow‐up of 6.6 years, we identified 638 case of kidney cancer. Table 2 showed SUA and the incidence of kidney cancer in the entire population. In brief, SUA levels were associated with an increased risk of kidney cancer incidence. After adjustment for potential confounders, participants with the top quartile of SUA had a 45% higher risk of kidney cancer as compared with those in the lowest quartile (HR1.45, 95%CI 1.08‐1.93). In addition, every 1 mg/dL increase in SUA level was associated with 13% increased risk of kidney cancer (HR 1.13, 95%CI 1.06‐1.21). In the subgroup analyses by gender, female in the top quartile of SUA had a 47% higher risk of kidney cancer as compared with those in the bottom quartile (HR1.47, 95%CI 1.01‐2.16), while we did not find sufficient evidence of association in men, although tests for interaction were not statistically significant (*P*‐interaction = 0.425; Table 3).

**TABLE 2:** Association between Serum uric acid and incidence of kidney cancer

	No of cases/ Person‐years	Hazard Ratio (95% Confidence Interval)			
		Age‐stratified model	Multivariable‐adjusted model 1^a^	Multivariable‐adjusted model 2^b^	Multivariable‐adjusted model 3^c^
Serum uric acid					
Quartile 1	80/730674	1.00	1.00	1.00	1.00
Quartile 2	135/727928	1.52(1.15, 2.00)**	1.33(1.00, 1.76)*	1.33(1.00, 1.76)*	1.20(0.91, 1.60)
Quartile 3	161/725440	1.75(1.34, 2.30)***	1.34(1.00, 1.78)*	1.34(1.01, 1.78)*	1.12(0.84, 1.49)
Quartile 4	262/724021	2.82(2.20, 3.63)***	1.93(1.46, 2.56)***	1.97(1.49, 2.60)***	1.45(1.08, 1.93)*
*P*‐trend		<.0001	<.0001	<.0001	.012
Continuous per unit increase(mg/dL)		1.33(1.26, 1.40)***	1.23(1.15, 1.30)***	1.23(1.16, 1.31)***	1.13(1.06, 1.21)***

^a^ Additionally adjusted for gender, education, ethnic, and index of multiple deprivation.

^b^ Additionally adjusted for alcohol consumption, smoking status, physical activity, and fruit and vegetable intake.

^c^ Additionally adjusted for BMI, comorbidities (diabetes, hypertension), medication (cholesterol lowering medication, blood pressure medication, NASIDs).

* 0.1 =< *P* < .5,

** 0.001 =< *P* < .01,

*** *P* < .001.

**TABLE 3:** Gender stratified hazard ratios for the risk of kidney cancer incidence serum uric acid

	Men		Women	
	No of cases/ Person‐years	Hazard ratio (95% Confidence Interval)	No of cases/ Person‐years	Hazard ratio (95% Confidence Interval)
Serum uric acid^a^				
Quartile 1	98/333430	1.00	41/394050	1.00
Quartile 2	79/335749	0.78(0.58, 1.05)	31/392339	0.68(0.43, 1.09)
Quartile 3	93/334994	0.87(0.65, 1.17)	55/393331	1.08(0.72, 1.62)
Quartile 4	145/334691	1.19(0.91, 1.56)	96/390379	1.47(1.01, 2.16)*
*P*‐trend		.104		.034
*P‐*interaction				.425
Continuous per unit increase (mg/dl)		1.08(0.99, 1.17)		1.18(1.05, 1.32)**
*P*‐interaction				.635

Note

Estimated effects were based on the fully adjusted model (see the footnote in Table 2).

^a^ The quartiles of different genders were calculated separately. In men: Q1 (89‐305 μmol/L), Q2 (305‐349 μmol/L), Q3 (349‐399 μmol/L), and Q4(399‐1068 μmol/L). In women: Q1 (89‐224 μmol/L), Q2 (224‐263 μmol/L), Q3(263‐308 μmol/L), and Q4 (308‐769 μmol/L).

* 0.1 =< *P* < .5,

** 0.001 =< *P* < .01,

*** *P* < .001.

### 3.2 Mortality of kidney cancer

We documented 188 kidney cancer deaths during a median follow‐up of 7.8 years. The age‐stratified HR of kidney mortality in the highest quartile of SUA was 2.49 (95% CI 1.61‐3.84) as compared to the lowest quartile. However, this association disappeared after further adjustment for other confounders (Table 4). In addition, we did not find apparent interaction effect in the subgroup analyses by gender (Table S1).

**TABLE 4:** Association between serum uric acid and mortality of kidney cancer

	No of cases/Person‐years	Hazard ratio (95% Confidence Interval)			
		Age‐stratified model	Multivariable‐adjusted model 1^a^	Multivariable‐adjusted model 2^b^	Multivariable‐adjusted model 3^c^
Serum uric acid					
Quartile 1	27/864087	1.00	1.00	1.00	1.00
Quartile 2	34/861137	1.08(0.65, 1.80)	0.83(0.49, 1.39)	0.83(0.49, 1.39)	0.78(0.46, 1.31)
Quartile 3	43/861797	1.30(0.80, 2.10)	0.78(0.46, 1.30)	0.79(0.47, 1.32)	0.70(0.42, 1.18)
Quartile 4	84/858812	2.49(1.61, 3.84)***	1.26(0.77, 2.05)	1.29(0.79, 2.10)	1.04(0.63, 1.72)
*P*‐trend		<.001	.112	.092	.470
Continuous per unit increase (mg/dL)		1.33(1.20, 1.46)***	1.15(1.03, 1.29)*	1.16(1.02, 1.30)*	1.09(0.96, 1.23)

^a^ Additionally adjusted for gender, education, ethnic, Index of multiple deprivation.

^b^ Additionally adjusted for alcohol consumption, smoking status, physical activity, and fruit and vegetable intake.

^c^ Additionally adjusted for BMI, comorbidities (diabetes, hypertension), medication (cholesterol lowering medication, blood pressure medication, NASIDs).

* 0.1 =< *P* < .5,

** 0.001 =< *P* < .01,

*** *P* < .001.

By excluding cases in the first 2 years of follow‐up, we still found a positive association between SUA and kidney cancer risk (Q4 vs Q1:HR 2.07, 95%CI 1.52‐2.82). When we excluded participants with renal failure and limited the analysis in people without gout, the association between SUA and kidney cancer was essentially unchanged (Table S2).

## 4 DISCUSSION

In this study of over 0.44 million participants, we found that SUA level was positively associated with the risks of kidney cancer. Moreover, in the subgroup analysis by gender, we only find sufficient evidence of association between SUA and kidney cancer incidence risk in women, but not in men. In addition, we did not find sufficient evidence that SUA was associated with kidney cancer mortality after adjustment for potential confounders.

For uric acid, our analyses found a positive association with the risk of kidney cancer. Similar results have been found in many retrospective and prospective studies of nonurological malignancies. In addition, various preclinical studies have showed that increased SUA may induce inflammatory stress responses, while elevated SUA stimulates various transcription factors that promote cell proliferation and migration. These changes help normal resting cells turn into highly aggressive cancer cells.^8^ In a study 493 281 individuals with a followed‐up time up to 25 years, Yiu et al observed that serum uric acid levels were associated with higher cancer risk, with a HR of 1.09 (95% CI: 1.06‐1.13) for each log increase in serum uric acid.^6^ Similarly, a meta‐analysis of 12 prospective studies of 632 472 subjects found that high SUA levels were correlated with an enhanced risk of total cancer (RR = 1.03, 95% CI 1.01 to 1.05).^9^ This study was the first large‐scale research identifying SUA as a positive predictor of kidney cancer incidence. We have confirmed these findings by noting that 1 mg/dL increment in SUA levels was associated with a 13% increased risk of kidney cancer incidence.

In the subgroup analysis by gender, we found sufficient evidence of an association between SUA and kidney cancer incidence risk in women, but not in men, although subgroup analysis did not show sufficient evidence of interaction effect. One explanation is that the case number was too small to test the effect of SUA on kidney cancer incidence risk in males. In addition, the effect of SUA on kidney cancer maybe smaller in males than in females, although subgroup analysis did not show a statistical significant interaction effect due to small case number. A similar gender differences in risk of renal cell carcinoma and occupational exposures to chlorinated aliphatic hydrocarbons was also reported in previous study.^10^ This may due to the differences in body fat content and metabolic activity between genders. More research is required to confirm the association in males.

The exact mechanism for the association between high SUA and kidney cancer is still unclear. One possible explanation is impaired renal excretion, high cell renewal status,^11^ and increased purine metabolism due to xanthine oxidase,^12^, ^13^ and elevated SUA levels, which is common in apoptosis. Cancer progression is also related to cell renewal and apoptosis. Other possible explanations for high levels of SUA are due to increased oxidative reactions on the presence of a tumor. It has been proved that reactive oxygen species (ROS) is involved in cell damage and cancer. Mitochondria, DNA, RNA, lipids, and proteins can be damaged by high levels of ROS. SUA reacts with ROS to be oxidized into allantoin, thereby avoiding the damage caused by ROS.^14^ However, increased SUA may also lead to impaired anticancer responses.^8^, ^15^ Therefore, due to the relationship between SUA and active oxidation response, high level of SUA may be a factor predicting the presence of tumors.

Many epidemiological studies have linked changes in SUA have to mortality of many nonurological cancer. However, few studies have investigated the association between SUA and renal cancer mortality. A retrospective analysis performed on 89 patients with renal cell carcinoma noted that an increase in SUA of more than 10% could predict metastasis (*P* < .0001).^16^ Another retrospective study confirmed that postoperative SUA can be used as a negative predictor of survival in patients with renal cell carcinoma.^17^ In our study, compared with the lowest quartile, the HR of kidney mortality stratified by age in the highest quartile of SUA was 2.49 (HR = 2.49, 95% CI:1.61‐3.84). However, these associations disappeared after further adjustment for other confounders. Future large size prospective researches are needed to confirm this finding.

A notable strength of our study is that this study was based on a nationwide prospective cohort, which collected detailed information on a wide range of covariates. This allowed us to adequately control potential confounders for the associations of interest. Other strengths include complete follow‐up, enough cases of kidney cancer incidence, and robust sensitivity analyses.

This study had several limitations. First, as an observational study, we cannot completely exclude residual confounding effects, so our results could not establish a causal relationship between SUA and kidney cancer. Second, since the SUA was measured only once, we could not assess the effect of alterations of SUA on kidney cancer over time. Third, the nonsignificant association between SUA and kidney cancer mortality may be due to the insufficient number of kidney cancer mortality cases. Fourth, given that serum urate is mainly excreted by the kidney, undiagnosed kidney cancer may influence kidney function and affect the SUA concentrations, so our findings might be confounded by reverse causation. However, further analyses by excluding cases during the first 2 years of follow‐up showed no major change in the primary results. Additionally, due to lack of histological information and fewer cases, analysis of the impact of SUA on kidney cancer subtypes was limited. Last, the association between SUA and the early stage kidney cancer incidence may be influenced by diagnostic bias. Participants with higher SUA may be more concern about their health and have more physical examinations, so they were more likely to be detected at the early stage if they suffered kidney cancer. We had no information about kidney cancer severity, so we cannot provide information about associations between SUA and kidney cancer severity.

In conclusion, SUA levels were positively associated with the occurrence of kidney cancer, especially in female subjects. In clinical and public health practice, women with high level of SUA deserve close surveillance for kidney cancer. Management for high SUA may have benefits in kidney cancer prevention. Future studies are required to confirm the causal relationship between SUA and kidney cancer and to investigate the underlying mechanisms.

## CONFLICT OF INTEREST

None declared.

## AUTHOR CONTRIBUTIONS

XD and JY conceived of the research. JY and QH analyzed the dataset. XD drafted the manuscript. XD, QH, ZJ, and JY revised the draft paper, read, and approved the final manuscript.

## Supporting information

Table S1‐S2

## Data Availability

UK Biobank is an open‐access resource. Bona fide researchers can apply to use the UK Biobank dataset by registering and applying at http://ukbiobank.ac.uk/register‐apply
