# Supplementary material for: Serum uric acid levels and risk of kidney cancer incidence and mortality: A prospective cohort study
Source: Cancer Med. 2020 Jun 14;9(15):5655–61. doi: 10.1002/cam4.3214 (PMC7402822; doi:10.1002/cam4.3214)
Supplement: Supplementary file 1 — Table S1‐S2 [file CAM4-9-5655-s001.docx]

**Supplemental file**

| **Table S1.** Gender stratified hazard ratios for the risk of kidney cancer mortality and serum uric acid | | | | | |
| --- | --- | --- | --- | --- | --- |
|  | Men | |  | Women | |
|  | No of cases/  Person-years | Hazard Ratio (95% Confidence Interval) |  | No of cases/  Person-years | Hazard Ratio (95% Confidence Interval) |
| Serum uric acid(mg/mg/dl) |  | 1.06(0.93, 1.22) |  |  | 1.21(0.95, 1.56) |
| *P-*interaction |  |  |  |  | 0.804 |
| Serum uric acid |  |  |  |  |  |
| Quartile 1 | 33/329201 | 1.00 |  | 20/385995 | 1.00 |
| Quartile 2 | 27/329149 | 0.82(0.49, 1.36) |  | 15/386273 | 0.60(0.23, 1.59) |
| Quartile 3 | 37/328712 | 1.06(0.66, 1.72) |  | 17/386706 | 0.91(0.39, 2.17) |
| Quartile 4 | 41/328994 | 1.02(0.63, 1.65) |  | 22/384908 | 1.25(0.55, 2.88) |
| *P*-trend |  | 0.679 |  |  | 0.354 |
| *P-*interaction |  |  |  |  | 0.674 |

Estimated effects were based on age-stratified model, with additional additionally adjustment for gender, education, ethnic, index of multiple deprivation, alcohol consumption, smoking status, physical activity, fruit and vegetable intake, BMI, comorbidities(diabetes, hypertension), and medication (cholesterol lowering medication, blood pressure medication, NASIDS)

| **Table S2.** Sensitivity analyses of serum uric acid and risk of kidney cancer incidence | | | | | | | | |
| --- | --- | --- | --- | --- | --- | --- | --- | --- |
|  | Men | |  | Women | |  | Whole | |
|  | No of cases/  Person-years | HR (95% CI) |  | No of cases/  Person-years | HR (95% CI) |  | No of cases/  Person-years | HR (95% CI) |
| Limiting the participants in people those with follow up above 2 years | | |  |  |  |  |  |  |
| Serum uric acid(mg/dl) |  | 1.08(0.98, 1.18) |  |  | 1.16(1.02, 1.33)* |  |  | 1.22(1.15, 1.31)*** |
| Serum uric acid |  |  |  |  |  |  |  |  |
| Quartile 1 | 78/332273 | 1.00 |  | 31/393352 | 1.00 |  | 60/729159 | 1.00 |
| Quartile 2 | 61/334826 | 0.75(0.53, 1.04) |  | 25/391509 | 0.76(0.43, 1.25) |  | 111/726015 | 1.49(1.08, 2.04)* |
| Quartile 3 | 71/333988 | 0.82(0.59, 1.13) |  | 44/392419 | 1.16(0.73, 1.85) |  | 123/723419 | 1.45(1.06, 1.99)* |
| Quartile 4 | 113/333594 | 1.13(0.83, 1.53) |  | 71/389248 | 1.43(1.00, 2.36) |  | 200/721715 | 2.07(1.52, 2.82)*** |
| Limiting the participants in people without renal failure | | |  |  |  |  |  |  |
| Serum uric acid(mg/dl) |  | 1.09(0.99, 1.18) |  |  | 1.18(1.05, 1.32)** |  |  | 1.22(1.15, 1.29)*** |
| Serum uric acid |  |  |  |  |  |  |  |  |
| Quartile 1 | 98/332964 | 1.00 |  | 41/393863 | 1.00 |  | 80/730206 | 1.00 |
| Quartile 2 | 77/335382 | 0.76(0.56, 1.03) |  | 31/392093 | 0.69(0.43, 1.59) |  | 135/727224 | 1.38(1.04, 1.82)* |
| Quartile 3 | 92/334620 | 0.87(0.65, 1.16) |  | 55/392948 | 1.09(0.73, 1.92) |  | 158/724474 | 1.43(1.09, 1.89)* |
| Quartile 4 | 140/333422 | 1.16(0.88, 1.52) |  | 95/389164 | 1.50(1.02, 2.19)* |  | 256/721652 | 2.05(1.57, 2.69)*** |
| Limiting the participants in in people without gout | | |  |  |  |  |  |  |
| Serum uric acid(mg/dl) |  | 1.06(0.97, 1.15) |  |  | 1.16(1.03, 1.31)* |  |  | 1.21(1.14, 1.29)^***^ |
| Serum uric acid |  |  |  |  |  |  |  |  |
| Quartile 1 | 94/323875 | 1.00 |  | 41/393595 | 1.00 |  | 78/726685 | 1.00 |
| Quartile 2 | 76/330078 | 0.77(0.56, 1.04) |  | 31/391958 | 0.69(0.43, 1.10) |  | 133/721290 | 1.4(1.05, 1.86)* |
| Quartile 3 | 91/329164 | 0.87(0.65, 1.17) |  | 55/392786 | 1.09(0.73, 1.64) |  | 156/717650 | 1.46(1.1, 1.93)** |
| Quartile 4 | 134/318580 | 1.15(0.87, 1.52) |  | 94/388934 | 1.48(1.01, 2.17)* |  | 249/702445 | 2.08(1.59, 2.74)*** |

*0.1=<p<0.5, **0.001 =< p < 0.01, *** p<0.001

Estimated effects were based on age-stratified model, with additional additionally adjustment for gender, education, ethnic, index of multiple deprivation, alcohol consumption, smoking status, physical activity, fruit and vegetable intake, BMI, comorbidities(diabetes, hypertension), and medication (cholesterol lowering medication, blood pressure medication, NASIDS)
